# Conditional autoencoder pre-training and optimization algorithms for personalized care of hemophiliac patients

**DOI:** 10.3389/frai.2023.1048010

**Published:** 2023-01-25

**Authors:** Cédric Buche, François Lasson, Sébastien Kerdelo

**Affiliations:** ^1^ENIB, Brest, France; ^2^IRL 2010, CNRS, Adelaide, SA, Australia

**Keywords:** deep learning, autoencoder, incremental optimization, conditional pre-training, global assays, hemophilia

## Abstract

This paper presents the use of deep conditional autoencoder to predict the effect of treatments for patients suffering from hemophiliac disorders. Conditional autoencoder is a semi-supervised model that learns an abstract representation of the data and provides conditional reconstruction capabilities. Such models are suited to problems with limited and/or partially observable data, common situation for data in medicine. Deep conditional autoencoders allow the representation of highly non-linear functions which makes them promising candidates. However, the optimization of parameters and hyperparameters is particularly complex. For parameter optimization, the classical approach of random initialization of weight matrices works well in the case of simple architectures, but is not feasible for deep architectures. For hyperparameter optimization of deep architectures, the classical cross-validation method is costly. In this article, we propose solutions using a conditional pre-training algorithm and incremental optimization strategies. Such solutions reduce the variance of the estimation process and enhances convergence of the learning algorithm. Our proposal is applied for personalized care of hemophiliac patients. Results show better performances than generative adversarial networks (baseline) and highlight the benefits of your contribution to predict the effect of treatments for patients.

## 1. Introduction

Medical errors are the third most common cause of death in the USA (Makary and Daniel, 2016). From an economic point of view, the annual international cost of these accidents is estimated to be 42 billion dollars (Harkanen et al., 2019). In view of these statistics, the World Health Organization advocates the development of measures to improve clinical decision making (Sheikh et al., 2017). The aim is to reduce such errors through the use of systems to assist in the individualization of therapies (Kawamoto et al., 2005). One method for individualization is to analyze the content of the patient's biological sample. In this way, biologists have sought to identify discriminating characteristics from biological results. However, although this approach is pertinent, it is limited by the lack of standardization of biological tests and consequent problems of reproducibility (Lasson et al., 2019). In order to fully exploit these sources of information, described as indicating patients' clinical phenotypes, a suitable approach is to extract highly abstract features using machine learning techniques.

According to the literature, deep models are capable of compactly representing highly non-linear functions (Bengio, 2009; Goodfellow et al., 2016), which makes them promising candidates. Moreover, unsupervised learning strategies such as autoencoders (AEs) are preferable in contexts where data are costly and only partially observable (LeCun et al., 2015), common situation for data in medicine. The conditional extension of AEs (CAE)s are also an interesting way to propose an independent preferential input to answer a problem of the type *p*(*y*|*x, z*). In the medical context *z* is a dose of medication, *x* is the initial biological result of the patient and *y* is the result of the test performed after the administration of the drug. This approach makes it possible to qualify the impact of drug administration. Conditional Variational Auto-Encoder (CVAE) has been proposed in the literature (Kingma et al., 2014; Sohn et al., 2015). It allows to control the generation such that *p*_*decoder*_(*x*|*h, c*) where c is a categorical or continuous variable representing a condition. Nevertheless, although these generative models have gained recent traction among the scientific community, they have not been developed to answer problems of the type *p*(*y*|*x, c*). Therefore, such approach is not relevant for your paper.

For these reasons, we focus on the use of a deep CAE (DCAE). More specifically, we will make proposals concerning the optimization procedure, which presents a two-sided difficulty. Firstly, the objective functions of these models are strongly non-convex functions with many critical regions. In order to avoid an impact on the convergence of the optimization methods, it is necessary to initialize the networks in a relevant way. Initialization strategies based on machine learning are suitable for this purpose. In a context of limited data, the unsupervised pre-training algorithm proves to be a relevant solution. Nevertheless, its operating principle is not adapted to CAEs, which is the first scientific hurdle we have to overcome. Secondly, a high degree of combinatorial complexity is associated with the hyperparametric optimization of networks composed of several hidden layers. Therefore, operational research strategies based on trial-and-error methods are of little relevance in the case of CAEs. To overcome this difficulty, the literature suggests dynamically adjusting the network parameters and hyperparameters. To this end, incremental learning and the random learning rate generation procedure ALRAO (Blier et al., 2018) can be used. However, the combination of these techniques has not yet been studied and the existing proposals for incremental CAEs are limited, which is the second scientific hurdle we face.

The motivation of this work is to improve personalized care for patients suffering from hemostasis disorders. In this context, this paper presents the use of DCAE to predict the effect of treatments. The contributions of this work are solutions to optimize the parameters and the hyper-parameters of DCAE. To do so, proposals describe conditional pre-training algorithm and incremental optimization strategies. This article is organized as follows. Section 2 presents the operating principle of CAEs and highlights the complexities of parametric and hyperparametric optimization. In Section 3, we detail our proposal, a conditional pre-training procedure for parametric optimization. In Sections 4, 5, we present our proposal of strategy based on incremental learning for hyperparameter optimization. Section 6 explains the application to the personalized management of hemophiliac patients. Finally, in Section 7, we review the results obtained and examine perspectives for future work.

## 2. State of the art

### 2.1. Autoencoders

An AE (Lecun and Soulie Fogelman, 1987) is a parametric model capable of extracting characteristic predicates from an unlabeled learning database (Géron, 2017). It is, therefore, an unsupervised model. An AE has two main parts: first an encoder function that maps the message (*x*) to a code, and second a decoder function that reconstructs the message (*r*) from the code. *h* represents the code (named the hidden layer). The dimension of the hidden layer *h*, means the size in the code layer, is a hyperparameter. The encoding and decoding functions should not be limited to trivial identity functions. In order to avoid this, it is necessary to constrain the dimensions of the hidden layer *h*. In this sense, there are two possible representations of the architecture of an AE. An AE is said to be undercomplete (Lecun and Soulie Fogelman, 1987) when the dimension of the hidden layer *h* is smaller than that of the input data *x* and overcomplete in other cases (Hinton et al., 2006; Vincent et al., 2008). Overcomplete AEs have additional properties, such as a decoding function robust to variations in *h* (Vincent et al., 2008), an encoding function able to withstand small perturbations in *x* (Rifai et al., 2011) or the ability to meet additional supervised criteria (Ng, 2017).

Autoencoders may be augmented in many different ways (Makhzani et al., 2016). It is common to append a one-hot label vector, *y*, to the inputs of the encoder and decoder. One interesting approach is CAEs (Makhzani et al., 2016). The aim of the learning phase is to minimize the hybrid cost function, which is defined by Equation (1). *J*_*reconstruction*_(θ) evaluates the unsupervised task (see Equation 2), *J*_*supervised*_(θ) quantifies the error associated with the conditional supervised criterion (see Equation 3 where ŷ corresponds to the prediction of *y*), and Ω(*h*) is the sparsity penalty (see Equation 4). Sparsity regularization methods seek to exploit the assumption that the output variable can be described by a reduced number of variables in the input space. It penalizes the objective function of the model, thus favoring the most relevant weights during the learning phase to answer the considered task. The parsimony penalty penalizes the absolute value of activations in layer *h* for observation *i*, scaled by a tuning parameter λ (Zhou et al., 2012a) and therefore regularizes the weights of a network. The value λ has a constant in this regularization term that needs to be treated as a hyperparameter.


(1) $$\begin{array}{l} J\left(\theta \right)=J_{reconstruction}\left(\theta \right)+J_{supervised}\left(\theta \right)+\Omega \left(h\right) \end{array}$$

(2) $$\begin{array}{l} J_{reconstruction}\left(\theta \right)=MSE\left(r,x\right) \end{array}$$

(3) $$\begin{array}{l} J_{supervised}\left(\theta \right)=MSE\left(ŷ,y\right) \end{array}$$

(4) $$\begin{array}{l} \Omega \left(h\right)=\lambda \underset{i}{\sum }|h\left(i\right)| \end{array}$$

Deep architectures are able to represent in a compact manner highly non-linear functions, which are difficult to represent by means of simple architectures (Hinton and Salakhutdinov, 2006; Bengio et al., 2007; Bengio, 2009). In such cases, the AEs are referred to as deep conditional autoencoders (DCAEs).

### 2.2. Optimization challenges

The learning phase of an AE aims to encode useful information in a neural network in a compact and distributed manner (Lecun and Soulie Fogelman, 1987). This implies defining the parameters and hyperparameters of the model.

#### 2.2.1. Parameters

Parametric optimization of deep AEs (DAEs) is a major difficulty. Unsupervised pre-training offers a relevant solution (Hinton et al., 2006). Using a greedy process, the parametric functions associated with the extraction of features from the distribution of the input data *p*(*x*) are iteratively initialized. This approach takes advantage of the simple AE optimization procedure. In the case of DCAEs, a conditional supervised criterion of type *p*(*y*|*x, c*) with {*x, c*} ∈ {ℝ^*n*^, ℝ} and *y* ∈ ℝ^*n*^ should be respected. By applying the unsupervised pre-training algorithm, we could then initialize the parameters associated with the extraction of characteristic predicates of *p*(*x*), i.e., those related to *p*_*encoder*_(*h*_*x*_|*x*) and *p*_*decoder*_(*x*|*h*_*x*_). The parameters of the supervised conditional criterion *p*_*supervised*_(*y*|*h*_*x*_, *c*) would be considered *a posteriori* to the initialization phase. The cost function *J*_*supervised*_(θ) risks leading to a bad convergence of the learning algorithm as the response to the supervised conditional criterion is achieved by a juxtaposition of hidden layers. The cost function *J*_*supervised*_(θ) is likely to have many critical regions. Therefore, the unsupervised pre-training algorithm does not seem to be a relevant initialization strategy in the case of DCAEs. So here we face a scientific obstacle.

#### 2.2.2. Hyperparameters

The hyperparametric optimization of deep models based on incremental learning (Fahlman and Lebiere, 1990) is relevant for data that can evolve over time. Also the ALRAO procedure (Blier et al., 2018) proves to be effective in assigning the learning rate to each node individually. This would make it possible to address this combinatorial complexity by means of a quasi autonomous strategy. Only two hyperparameters would then have to be defined: the minimum and maximum values of the learning rate. However, all the various methods we have just outlined have limitations. Indeed, the proposal of Zhou et al. (2012a), which makes it possible to dynamically define deep architectures in static database contexts, implies the optimization of many hyperparameters and proves to be sensitive to the initial dimensions of the hidden layers. Conversely, the non-parametric approach of Pratama et al. (2018), which presents itself as an efficient solution for optimizing simple DAEs on continuous streams of data, is not applicable to cases of deep architectures and is specific to e-learning (Ashfahani et al., 2020). Moreover, in order to eliminate the problem of overfitting encountered in the ALRAO proposal (Blier et al., 2018), it could be compatible to use it in combination with incremental learning. This would make it possible to promote the pruning of networks in order to remove unnecessary parameters without impairing performance (LeCun et al., 1990; Reed, 1993). Although the various theoretical concepts associated with these proposals are relevant, none of them is a turnkey solution. Therefore, we are faced another scientific obstacle.

## 3. Parametric optimization

In this section, we present our proposed conditional pre-training algorithm to address the complexity of parametric DCAE optimization.

**Figure 1:**
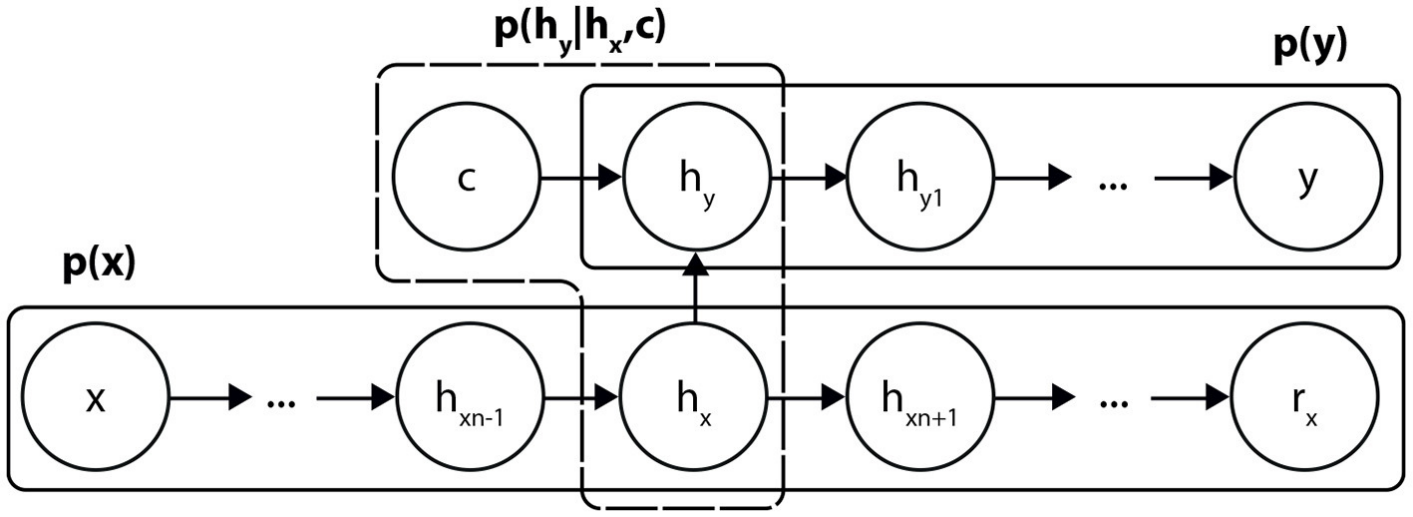
Breakdown of the DCAE into three sub-architectures.

The procedure for optimizing simple AEs is efficient and does not pose any difficulties. To take advantage of this, we split the architecture of the DCAE into three basic building blocks (Figure 1). Two of these are associated with the distributions of the input *p*(*x*) and output data *p*(*y*) while the third corresponds to the supervised conditional link *p*(*h*_*y*_|*h*_*x*_, *c*), where *h*_*x*_ and *h*_*y*_ are the compact representations of *x* and *y*.

**Figure 2:**
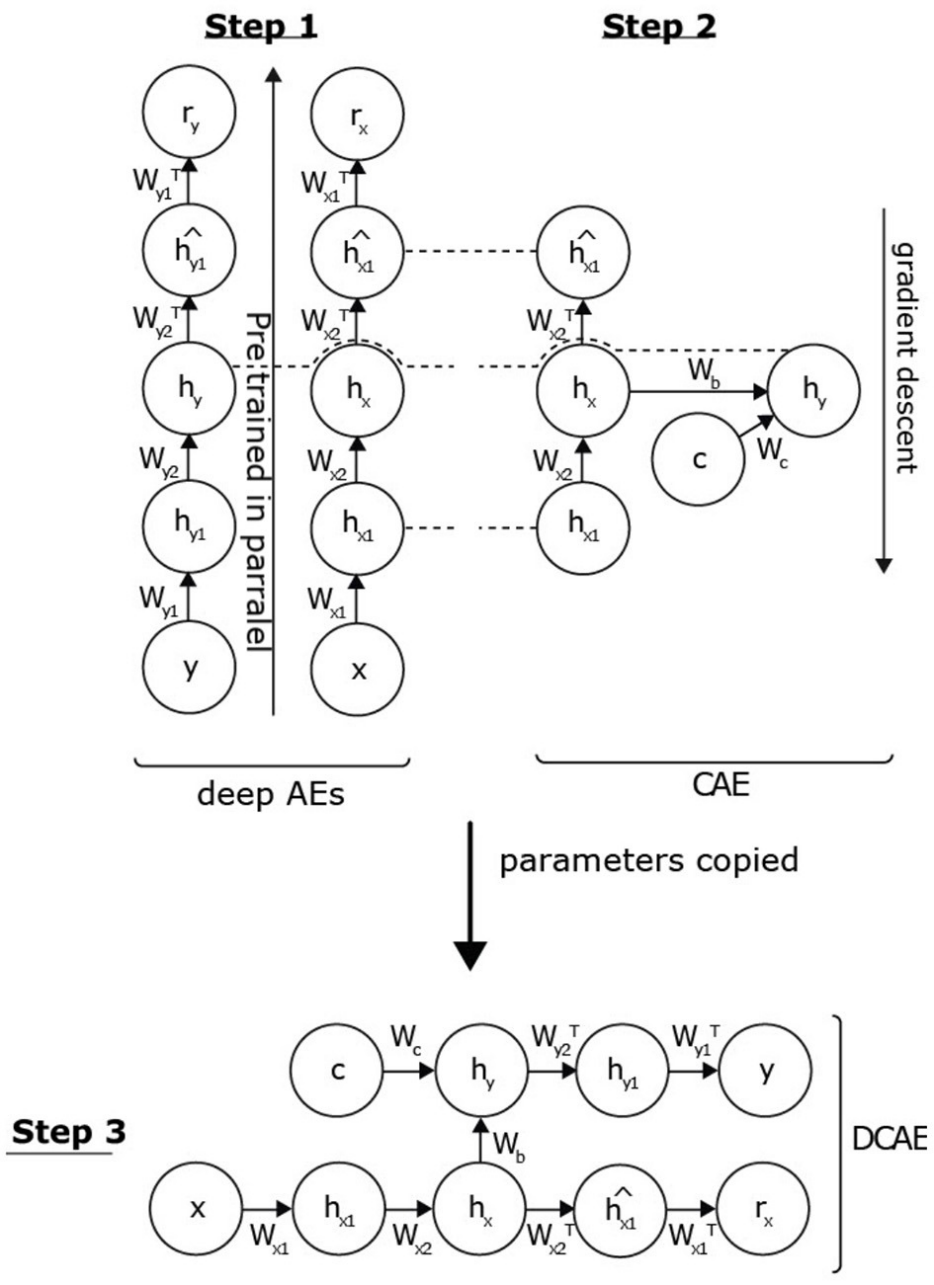
Conditional pre-training strategy. First, we treat the parametric functions associated with the extraction of features of the data distributions *p*(*x*) and *p*(*y*), considering them as two deep AEs. As previously pointed out, it is possible to initialize these networks by learning by applying the unsupervised pre-training algorithm. Being independent, these two models can be processed in parallel (step 1: parallel pre-training). After this first step, we are able to extract the probabilities of the hidden layers *h*_*y*_ and *h*_*x*1_ by inference. The initialization of the parameters associated with the supervised conditional link is then performed through an CAE trained to answer the problem *p*(*h*_*y*_|*h*_*x*1_, *c*) (step 2: supervised conditional link). These two successive steps initialize all the hidden layers of the DCAE. It is then necessary to then export the various weight matrices within the deep architecture in order to jointly adjust all the parameters of the network (step 3: copying of the parameters).

We propose to initialize the DCAE by adopting a greedy strategy (see Figure 2). First, we treat the parametric functions associated with feature extraction of the data distributions *p*(*x*) and *p*(*y*), considering them as two DAEs. It is possible to initialize these networks by applying the unsupervised pre-training algorithm (Hinton et al., 2006) (step 1). Next, the initialization of the parameters (*W*_*b*_ and *W*_*c*_) associated with the supervised conditional link is performed. *W* is the weight matrix. A CAE links the two AEs to the condition *c* (step 2). Following this pre-training, the parameters of the AEs and CAEs are copied to initialize the DCAE, which will, in turn, be trained in a conditionally supervised way (step 3). We detail each of these steps below.

### 3.1. Step 1: Parallel pre-training

**Figure 3:**
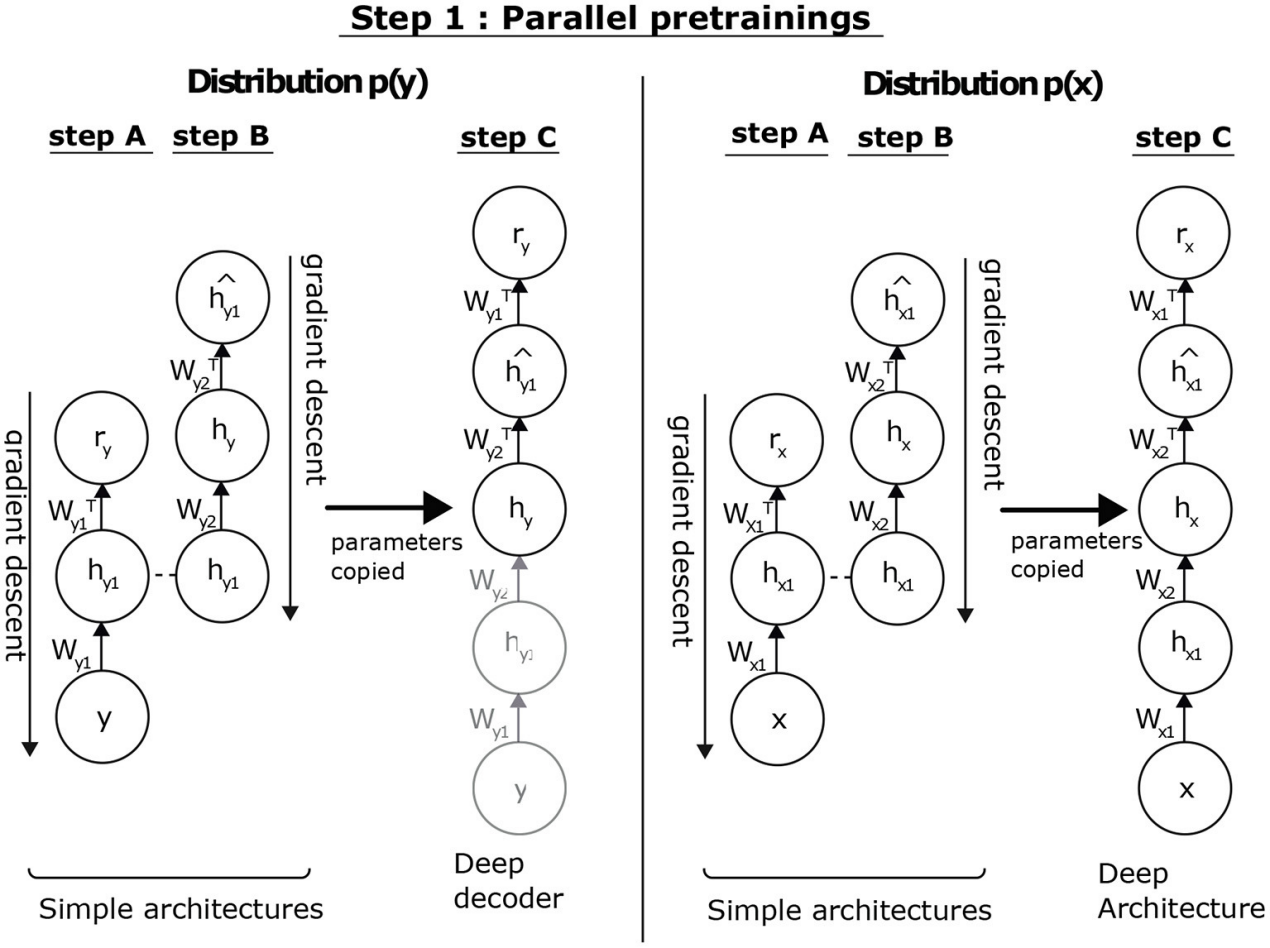
Parallel pre-training step. Concerning *p*(*x*), we aim at initializing the parameters associated to *p*_*encoder*_(*h*_*x*_|*x*) and *p*_*decoder*_(*x*|*h*_*x*_). To do so, we have previously shown that it is possible to apply the unsupervised pre-training algorithm. Concerning *p*(*y*), it is the parameters associated with *p*_*decoder*_(*y*|*h*_*y*_) that we seek to initialize in a consistent manner. To this end, we consider defining a deep AE of the same dimension, whose goal is to extract the *h*_*y*_ representation of the data *y*. Reasoning by analogy, the unsupervised pre-training of this model is sufficient to obtain the values values. This approach allows us to initialize the two sub-architectures associated to *p*(*x*) and *p*(*y*). By inference, we can then deduce the distributions *p*_*encoder*_(*h*_*x*_|*x*) and *p*_*encoder*_(*h*_*y*_|*y*).

The distributions of the input and output data are independent, so we can process them in parallel. *p*(*x*) aims to initialize the parameters associated with *p*_*encoder*_(*h*_*x*_|*x*) and *p*_*decoder*_(*x*|*h*_*x*_). We can apply the unsupervised pre-training algorithm (Hinton et al., 2006). In the case of *p*(*y*), it is the parameters associated with *p*_*decoder*_(*y*|*h*_*y*_) that we wish to initialize in a consistent manner. This consists in defining a DAE of the same dimension, with the aim of extracting the *h*_*y*_ representation from the *y* data. By reasoning by analogy, the unsupervised pre-training of this model is sufficient to obtain the desired values. As illustrated in Figure 3, this approach allows us to initialize the two sub-architectures associated with *p*(*x*) and *p*(*y*). By inference, we can then deduce the distributions *p*_*encoder*_(*h*_*x*_|*x*) and *p*_*encoder*_(*h*_*y*_|*y*).

#### 3.1.1. Step 2: Supervised conditional link

Following this first step, the parameters associated with the conditional supervised link should now be considered. In the architecture of the DCAE, it is not conceivable to initialize them randomly. This would generate a break in the gradient descent chain, which would cancel the initialization of the parameters associated with *p*_*decoder*_(*y*|*h*_*y*_). We can consider a CAE whose goal is to answer the problem *p*(*h*_*y*_|*h*_*xn*−1_, *c*), where *h*_*xn*−1_ is the layer before *h*_*x*_. At the end of the parallel pre-training stage, we know the distributions of the hidden layers *h*_*xn*−1_, *h*_*y*_ as well as the parameters associated with the reconstruction of *h*_*xn*−1_. It is then necessary to randomly initialize the other parameters of this simple architecture before applying the gradient descent algorithm. The compact representation *h*_*x*_ is then adjusted during the learning phase to acquire the additional properties necessary to meet the conditional supervised criterion.

#### 3.1.2. Step 3: Parameter adjustment

The previous steps encode the useful information in the initial parameter distribution, it is then necessary to continue training the model by applying the learning algorithm. In this phase, the parameters of the three previously considered sub-architectures will be adjusted jointly. The cost function of the DCAE is presented in Equation (1). Nevertheless, given the properties of the unsupervised pre-training strategy, it is possible to cancel the regularization term Ω(*h*) present in the latter (Equation 5). Indeed, the study by Erhan et al. (2010) presents this initialization technique as a form of regularization in its own right. As indicated in Rifai et al. (2011) and Goodfellow et al. (2016), taking into account sparsity penalties during the pre-training phase proves to be a sufficient condition for obtaining a regularized deep model. Such property has been supported by experiments described in Lasson (2020, 46) and is inline with analysis made by Rifai et al. (2011) and Goodfellow et al. (2016).


(5) $$\begin{array}{l} J\left(\theta \right)=J_{reconstruction}\left(\theta \right)+J_{supervised}\left(\theta \right) \end{array}$$

## 4. Hyperparametric optimization for AEs

Hyperparametric optimization can be achieved by jointly and dynamically adjusting the parameters and hyperparameters of networks. In this section we will consider simple incremental AEs. Section 5 will use the results obtained on a simple AE for cases of DAEs and DCAEs. A schematic overview of the operating principle of our proposal is shown in Algorithm 1.

**Algorithm 1:**
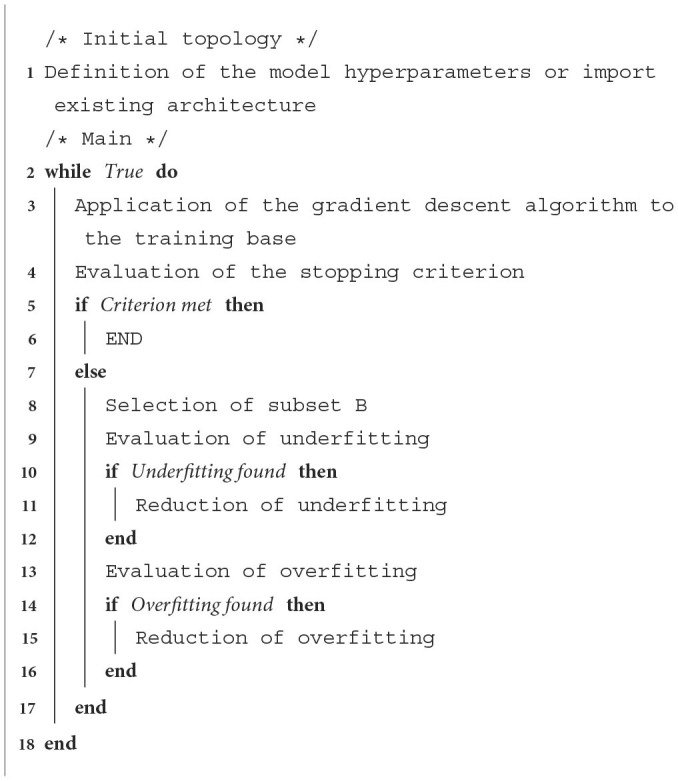
Simple incremental AE.

### 4.1. Initialization

Incremental learning involves defining an initial situation. It is a matter of initializing the size of its hidden layer and its learning rates. Two approaches can be considered for this stage, one consisting of manual initialization by the user, the other based on the reuse of a previously trained model. Whichever approach is used, it is necessary to complete this initialization stage by applying the gradient descent algorithm to the entire learning base.

In the first case, it is important to note that the initial size of the hidden layer will influence the convergence speed of the algorithm. In order to avoid the need for a large number of iterations to solve these optimization problems, it is possible to use more sophisticated strategies of adding and removing hidden units than those proposed by Pratama et al. (2018). Underpinned by the assumption that the performance of an over-fitted model can be achieved by a narrower architecture (LeCun et al., 1990), we will favor the definition of overcomplete architectures during this initialization phase. We will, therefore, have to develop a pruning technique capable of efficiently removing superfluous information from the networks. For the definition of the learning rates, we will use the random generation procedure ALRAO. It will then be necessary to carry out a grid search during this initialization stage to determine the order of magnitude of the two hyperparameters that this method integrates (maximum and minimum learning rate). Furthermore, we will also have to define the type of regularization we wish to use. In their work, Zhou et al. (2012a) and Pratama et al. (2018) used DAEs. We will study the applicability of this incremental strategy to the cases of DAE, sparse AE and unregularized AE.

In the second case, we wish to perform parametric and hyperparametric optimization of a model that has been trained upstream. Through this approach, we will be able to incrementally enrich its network to adapt it to a similar context, i.e., to achieve unsupervised learning by transfer or domain adaptation.

#### 4.1.1. Stopping criteria

As a stopping criterion, Zhou et al. (2012a) propose to confront the cardinal of the set of poorly discriminated training samples with a threshold (τ). This approach implies the optimization of a new hyperparameter. In a continuous learning context, it could be seen as an expectation criterion. Its value would then be allocated to the size of the mini-batches used by the learning algorithm. In a static database context, however, its value is correlated with the quantity and quality of the training samples. It is then necessary to optimize it by means of an operational research using the generalization error obtained from the learning phase. In other words, the adjustment of this value implies running this incremental protocol several times.

In order to develop a quasi-autonomous optimization strategy whose aim is to obtain models that neither overfit nor underfit: the role of the stopping criterion is to stop the algorithm once the objective has been reached. Without it, the joint parameter adjustment step would be repeated in a loop until overfitting occurs. The hidden units removal step would then take over, with the risk of degrading the model's performance. In order to avoid such a situation, we will have to check the impact of this pruning on the model's performance. In the event that performance will decreased, the units considered will have to be retained. Moreover, it will also be necessary to stop the algorithm before the appearance of this divergence. To do this, we propose to use the early stopping principle (Prechelt, 1996). Therefore we will have to estimate the generalization error during iterations on an additional validation set.

#### 4.1.2. Automatic selection of the subset

For the selection of the subset of poorly discriminated training data (denoted *B*), the convergence of the learning algorithm must be taken into account. To do this, we will use the value of the cost function *J*(θ). The k-means data partitioning algorithm can separate the training samples into two subsets without using a threshold value. However, this algorithm turns out to be highly sensitive to its initialization (choice of centroids). Therefore, we will proceed in a similar way to Zhou et al. (2012b), using the expectation of *J*(θ) as a threshold value. This indicator is a relevant solution for selecting the subset *B* as it is highly sensitive to extreme values.

#### 4.1.3. Reducing underfitting

Although overfitting can be effectively addressed through pruning techniques, the number of hidden units needed to reduce underfitting is complex to determine. Therefore, we will proceed in a similar way to Pratama et al. (2018) adding a maximum of one unit per iteration. The idea is to assign the opposite value of the residual error rate to the parameters of this new unit. In addition to the fact that it is not compatible with mini-batch learning, the application of the latter to a static database context would induce a high sensitivity to outliers. Therefore, we propose to optimize these parameters on the subset *B* by applying the gradient descent algorithm (on a single epoch). During this stage, the other parameters of the model will be frozen. As we wish to use the ALRAO procedure, the learning rate associated with this new unit will be initialized *via* a pseudo-random number generator.

#### 4.1.4. Reducing overfitting

It is possible to use more advanced pruning strategies than those put forward by Zhou et al. (2012b) and Pratama et al. (2018). The former allows the removal of a maximum of one unit per iteration, while the latter is not able to limit the influence of the initial topology. Therefore, we propose to use a non-parametric strategy based on a data partitioning method called affinity propagation (Frey and Dueck, 2007). This iterative algorithm is based on a principle of sharing similarities, called affinities, with the aim of building a tree between observations considered similar (Frey and Dueck, 2007). Unlike the k-means algorithm, the affinity propagation automatically determines the number of classes present in the observation set. Applied to our context, it would allow us to select the subset of hidden units with the lowest degrees of activation. We would then have 1 ≤ Δ*M* ≤ (*L* − 1), where Δ*M* is the number of units to be deleted and *L* the width of the hidden layer. In order to avoid an unintentional degradation of the model's performance, we will have to estimate the generalization error *a priori* and *a posteriori* of unit deletion. This pruning step will be validated only if it is beneficial to the network.

#### 4.1.5. Conditional extension

Because of its working principle, our proposal can also be applied to the case of a CAE. Indeed, the probability that the hidden layer of this model is equal to *p*_*encoder*_(*h*|*x*). It is, therefore, independent of the conditional variable and so we will use the unsupervised task *p*_*decoder*_(*x*|*h*) to estimate the presence of overfitting or underfitting. The only difference will be in the steps of evaluating the stopping criterion and selecting the subset *B*, which will use the hybrid cost function presented in Equation (1).

## 5. Hyperparametric optimization for DAEs and DCAEs

The unsupervised pre-training algorithm is a relevant solution for dealing with the strongly non-convex cost functions of DAEs (Hinton et al., 2006). Also, it is less costly to independently optimize a series of *n* single AEs than to deal jointly with the *n* hidden layers of a DAE (Rifai et al., 2011). In other words, pre-training can also be used to reduce the combinatorial complexity that is associated with the hyperparametric optimization of these models. Therefore, we propose to dynamically define the DAE topology using a greedy algorithm similar to the unsupervised pre-training method. To do so, we will consider simple incremental AE as building blocks. The working principle of our proposal, which we have named the unsupervised incremental optimization strategy, is illustrated in Algorithm 2.

**Algorithm 2:**
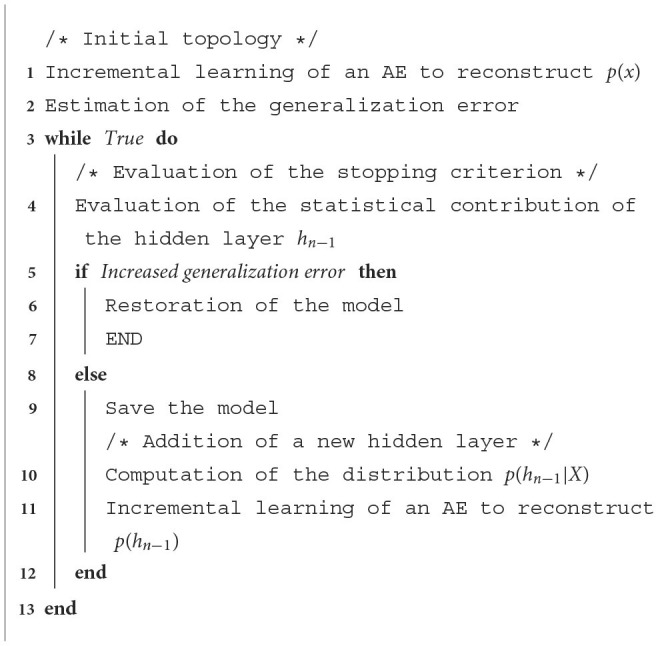
Unsupervised incremental optimization.

### 5.1. Initial topology

In order to define the initial situation of this procedure, we propose to optimize a single incremental AE. The initial topology of this simple model can then be specified through the two approaches previously described, namely by the user or by reusing a previously trained model. Following this definition, this quasi-autonomous model needs to be optimized before estimating its generalization error on an additional validation set.

### 5.2. Evaluation of the stopping criterion

With the objective of adjusting the parameters and hyperparameters of a deep architecture incrementally, we need to define a stopping criterion for our algorithm. Hence, we propose to evaluate the statistical contribution of the hidden units that have been optimized in the last iteration. The parameters of all the layers are then exported into a single architecture and jointly optimized by applying the learning algorithm. The generalization error of the resulting model is then estimated and compared with the errors of the previous iterations. When a decrease in the error rate is observed, the values of these parameters and hyperparameters are saved. Otherwise, the contribution of the central layer is deemed negative. The algorithm is then stopped and the previously saved values restored.

### 5.3. Addition of a new hidden layer

To increase the degree of abstraction of the model, we propose to proceed in a similar way to classical pre-training. For this purpose, the distribution of the hidden layer of the previously considered block is obtained by applying the encoding functions. It is then used during the next iteration to optimize a new incremental AE.

### 5.4. Application to DCAEs

Given the conditional pre-training algorithm that we proposed, we are able to adapt this optimization strategy to the case of DCAEs. Our first intention was to follow its operating principle as closely as possible. To do this, we proposed to optimize in parallel the two DAEs associated with the distributions *p*(*x*) and *p*(*y*) by adopting the strategy shown in Algorithm 2. Then, after obtaining the compact representations by applying the encoding functions, we would have optimized the parameters and hyperparameters of the supervised conditional link *via* an incremental CAE. The gradient descent algorithm would then have been applied in order to jointly adjust the set of weight matrices that compose the DCAE thus obtained. However, by this approach, the hyperparameters of the DAEs associated with the distributions *p*(*x*) and *p*(*y*) are optimized through our unsupervised incremental proposal. Therefore, the cost function *J*(θ) of the DCAE is not considered when evaluating the statistical contribution of these various hidden layers. In order to remedy this, i.e., to take into account the function *y* = f^*^(*x, c*) to be approximated during this optimization phase, we considered a second protocol. This method, which we have named the conditional incremental optimization strategy, is illustrated in Algorithm 3.

**Algorithm 3:**
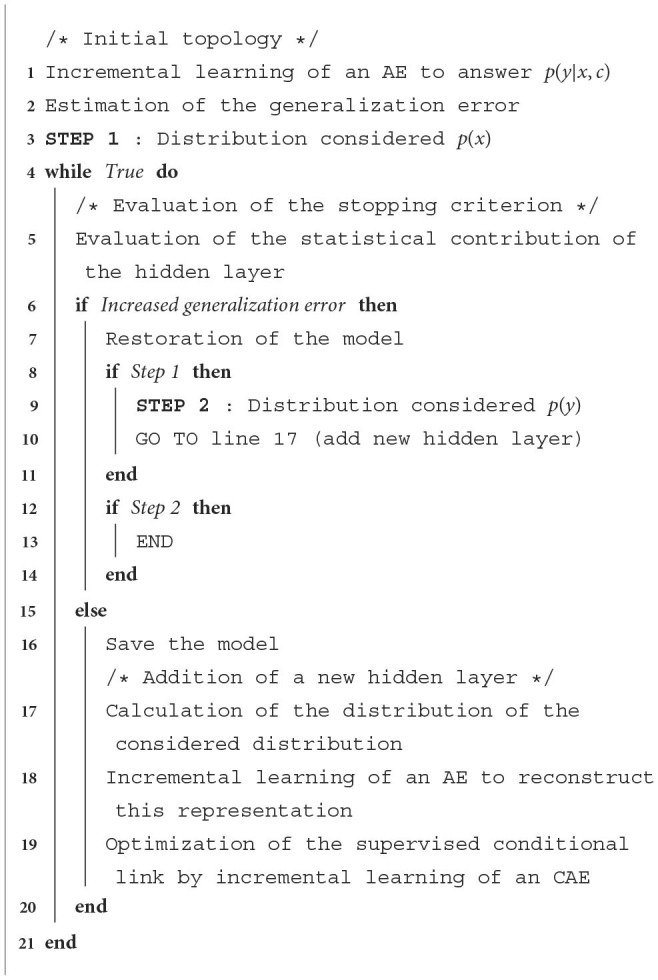
Conditional incremental optimization.

First, we need to evaluate the performance of a simple architecture whose objective is to complete the supervised conditional task *p*(*y*|*x, c*). We propose to train a CAE incrementally before estimating its generalization error on an additional validation set. In order to ensure the relevance of this hyperparametric optimization, we consider the DCAE cost function when evaluating the stopping criterion. To this end, we reconsidered the operating principle of conditional pre-training in order to deal with the distributions *p*(*x*) and *p*(*y*) in a sequential manner. In the first step, we propose to define the deep architecture that is associated with the input data. At each iteration, an incremental AE is then trained to reconstruct the compact representation of *p*(*x*) and then an incremental CAE is trained in turn to answer the problem *p*(*y*|*h*_*x*_, *c*). In order to evaluate the statistical contribution of this new hidden layer *h*_*x*_, the set of parameters associated with the reconstruction of *p*(*x*) and the supervised link is then exported within a DCAE. By applying the gradient descent algorithm, the dynamics of the generalization error of this deep conditional model is then estimated on an additional validation set. When an improvement of the performances is obtained, the model thus obtained is saved. Otherwise, this new hidden layer should be rejected. The previous version of the DCAE is then restored. The second step is to repeat this protocol for the *p*(*y*) distribution. Incremental CAEs are then iteratively trained to answer the problem *p*(*h*_*y*_|*h*_*x*_, *c*) until the stopping criterion is met, *h*_*y*_ being the compact representation of *y*.

## 6. Application

In this section, we present the application to predict the minimal dose of medication sufficient to reduce the risk of bleeding or thrombosis in a patient with a hemostasis disorder. The stakes of such an optimization are the improved quality of care and therapeutic results, plus decreased treatment costs.

### 6.1. Biological proposal

The problem can be modeled as a regression of type *p*(*y*|*x*) where *x* is the result of the patient's biological test and *y* is the optimal dose of drug to administer. Usually, the answer to such a problem is a function *y* = *f*(*x*) where the rule *f* is derived from expert knowledge. It could, therefore, be established based on data from sick patients, receiving treatment or not, for which the {*x, y*} pairs would have been determined by clinical evaluations. However, in view of the high temporal and economic costs necessary for creating a specific cohort and generating these data, this approach cannot be taken. To circumvent this need for clinical assessments, we considered taking advantage of the predictive nature of biological tests. Therefore, we propose here to qualify the impact that administering a drug could have on the patient's overall test result. This is a conditional model whose aim is to complete a task of type *p*(*y*|*x, c*), where *c* is a drug dose, *x* is the initial biological result of the patient and *y* is the result of the test performed after the drug administration.

### 6.2. Dataset

**Figure 4:**
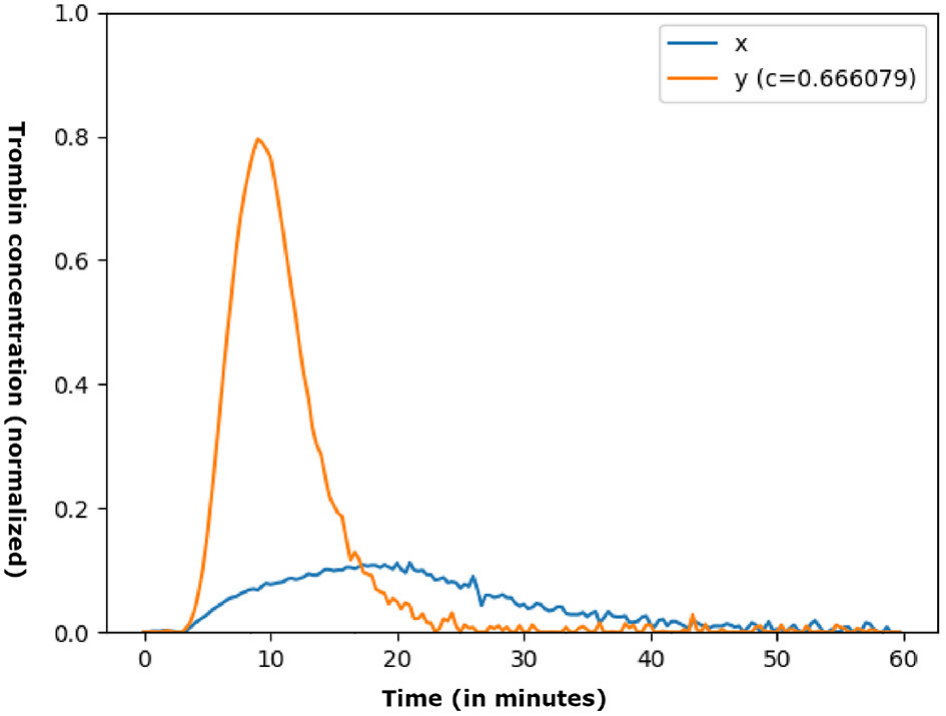
Example of {*x, c, y*} data. The abscissa represents the measurement time while the ordinate illustrates the concentration of thrombin (normalized). The dose is expressed over the whole [0,1], *c*≈0.67 corresponds to the injection of a concentration of 83.62% of therapeutic factor VIII.

The dataset was constructed by *in vitro* simulation of 115 plasmas from severe hemophilia A patients without inhibitors, for which increasing doses of therapeutic factor VIII concentrates were artificially injected. It was thus composed of 1992 triplets of {*x, c, y*} data in which, *x* is the result of the thrombosis generation test (GT) of a simulated patient, *c* is a dose of therapeutic factor VIII and *y* is the thrombinogram (evolution of thrombin concentration) resulting from the injection. Of these triplets, 1,297 were associated with the training set (73 plasmas), 325 with the validation set (19 plasmas), while the remaining 370 were reserved for the test base (23 plasmas). Figure 4 shows an example of a triplet. In this case, we have 180 measurements in 60 min (three concentration measurements per minute). Therefore, dimensions of *x* and *y* are 180 and *c* is a value.

### 6.3. Baseline

**Figure 5:**
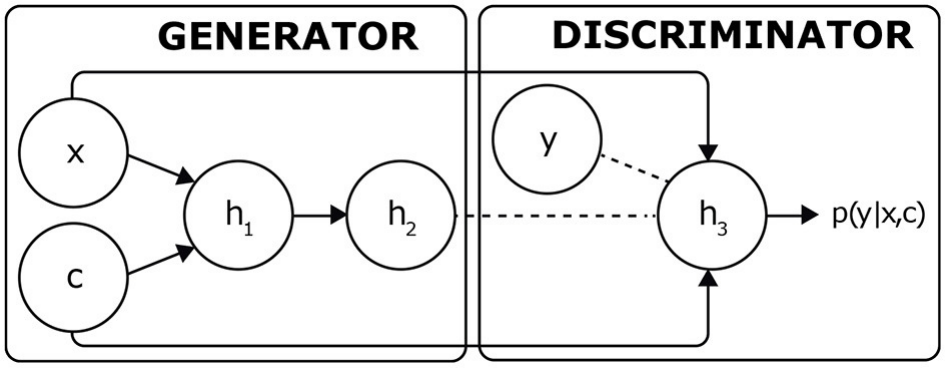
Application of GANs to a task of type *p*(*y*|*x, c*). By interacting with the discriminant, the generator is trained to reconstruct the output datum *y* *via* the synthetic datum *h*_2_.

Generative adversarial networks (GANs) are generative models that were proposed in response to problems of increasing database size. In our case study, GANs are used as reference models, applying them to a conditional problem of type *p*(*y*|*x, c*) where *c* is a dose of factor VIII, *x* is the initial thrombinogram of the patient and *y* is the thrombinogram of the GAN test carried out after the injection. It would, therefore, be appropriate to employ conditional GANs (Mirza and Osindero, 2014): an extension is presented in Figure 5, which aims to constrain the inputs of these two networks by adding visible units.

### 6.4. Objectives

#### 6.4.1. Robustness

We will compare the convergence of our proposal with that of a more classical strategy. By the mean values and standard deviations of the reconstruction errors, we will ascertain the robustness of the incremental CAE and, therefore, our contribution to the hyperparametric optimization of simple conditional architectures.

#### 6.4.2. Parameter optimization

Following this first evaluation, we will compare the average error rates obtained by the conditional pre-training with those resulting from a random initialization by the heuristic of Glorot and Bengio (2010), i.e., by pitting our proposal against the only feasible initialization method available in the literature.

#### 6.4.3. Hyperparameter optimization

It will then be necessary to evaluate the convergence of our conditional incremental optimization strategy, a proposal we have developed in response to the dual difficulty of optimizing these deep architectures.

#### 6.4.4. CAE vs. GAN

Finally, we will compare the reconstruction errors thus obtained with those of a reference model: the conditional GAN. This evaluation will allow us to justify our initial choice for the DAEs and, thus, to underline their interest for systems to assist individualization of therapies.

#### 6.4.5. Metrics

For thrombinograms, the peak height (maximum amplitude) is inversely proportional to the severity of the hemophilia. So, in view of the constitution of the databases considered, it seems appropriate to estimate the reconstruction error by means of a normalized square error. We will use the square root of the normalized root mean square error (NRMSE). This metric, which is defined in Equation 6 (where *x* is a vector, $\hat{x}$ is the prediction of *x*, *n* the dimension of *x*), will also be used in the objective functions of the previous models.


(6) $$\begin{array}{l} NRMSE=\frac{1}{n}\frac{\sqrt{\overset{n}{\underset{i=1}{\sum }}{\left(x_{i}-{\hat{x}}_{i}\right)}^{2}}}{max\left(x\right)-min\left(x\right)} \end{array}$$

### 6.5. Experimental method

We will first deal with the case of simple architectures by taking the following two approaches:

- Approach n.^o^ 1: application of an incremental CAE to the considered database.^1^ The duration of its optimization will be denoted duration_n.^o^1_;
- Approach n.^o^ 2: hyperparametric optimization of a CAE *via* a random search.^2^ The duration of the latter will be limited to the duration_n.^o^1_.

In order to study the convergence of these two optimization approaches, they will both be rerun ten times. Following this first evaluation, we will focus on the case of DCAEs by considering the following three optimization strategies:

- Approach n.^o^ 3: Use of our conditional incremental optimization proposal (see text footnote 1). The duration of its application will be denoted duration_n.^o^3_;
- Approach n.^o^ 4: hyperparametric optimization of a DCAE performed through a random search^3^ limited to the duration_n.^o^3_. The parameters of this model will be initialized by the heuristic of Glorot and Bengio (2010) and optimized with respect to the regularized objective function (see Equation 1).
- Approach n.^o^ 5: hyperparameter optimization of a DCAE performed through a random search (see text footnote 3) limited to the duration_n.^o^3_. The parameters of this model will be initialized by our proposal of conditional pre-training and then optimized with respect to the unregularized objective function (see Equation 5).

In order to study the large combinatorial complexity of hyperparametric optimization of DCAEs and the convergence of our proposals, these three approaches will also be rerun 10 times. Moreover, in order to justify our initial choice of DAEs, we will try to apply GANs to this conditional task. Given the specificity and complexity of their learning procedure, it would not be relevant to adopt a hyperparametric optimization strategy with a time constraint. Therefore, a progressive adjustment of the topologies will be made through various grid searches.^4^

### 6.6. Results

Experiments were made using the following programming libraries and hardware configuration:

- Memory: 15 GiB
- Processor: Intel®Core™I7-6700 CPU@3.40 GHz x8
- Python: 2.7.12
- Tensorflow: 1.8.0
- Numpy: 1.16.6

#### 6.6.1. Simple architectures

**Figure 6:**
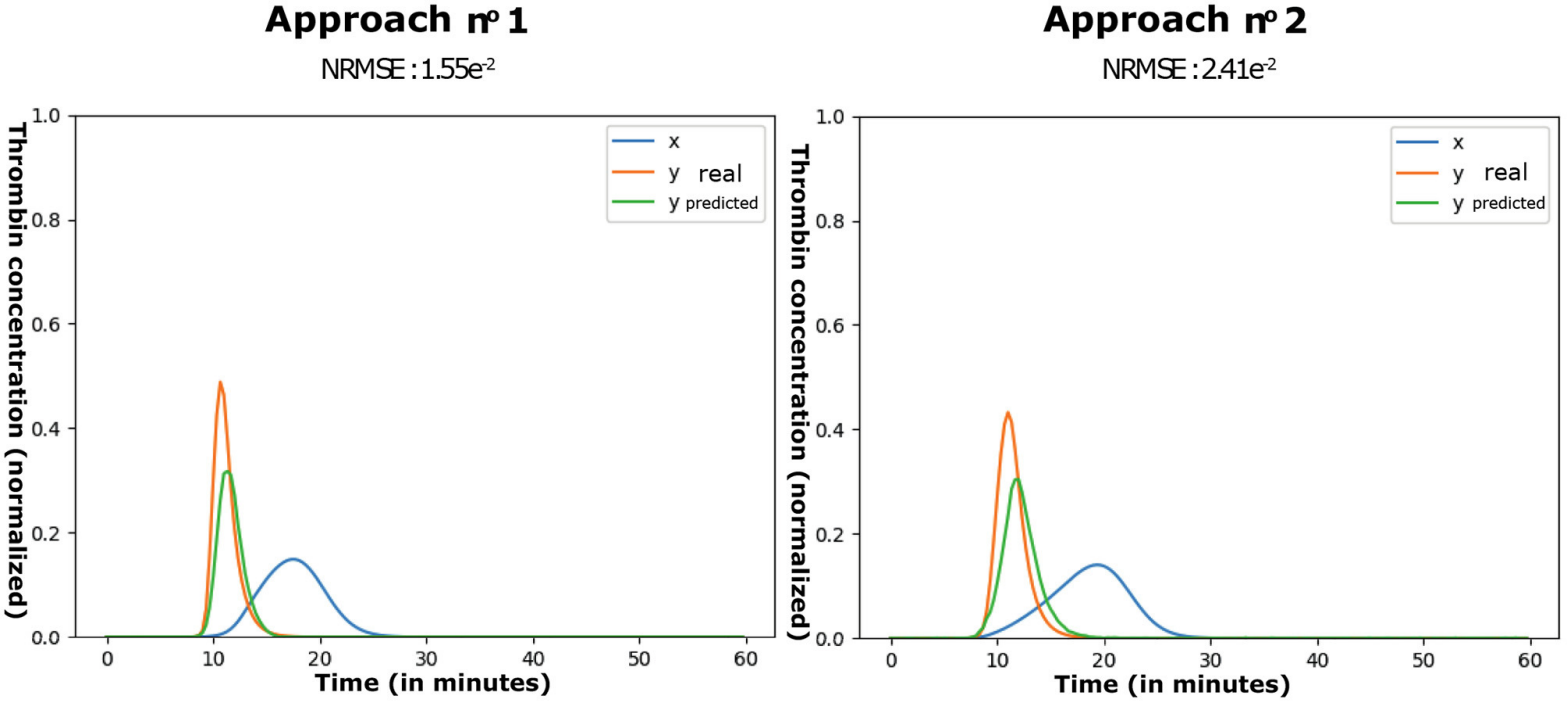
Examples of predictions by approaches n.^o^ 1 and 2.

The application of the incremental CAE optimization procedure (approach n.^o^ 1) on the *in vitro* database took 730s on average. Considering the ten retries performed, 324 CAE were optimized through approach n.^o^ 2. The associated reconstruction error rates (NRMSE) are shown in Table 1 and illustrated in Figure 6.

**Table 1:** Performances obtained on the *in vitro* database.

**Approach**	**Learning error**	**Test error**
n.^o^ 1	9.03*e*^−2^ ±5.54*e*^−4^	9.35*e*^−2^ ±4.61*e*^−4^
n.^o^ 2	1.49*e*^−1^ ±6.42*e*^−2^	1.50*e*^−1^ ±7.04*e*^−2^
n.^o^ 3	6.19*e*^−2^ ±7.41*e*^−4^	6.11*e*^−2^ ±6.13*e*^−4^
n.^o^ 4	9.25*e*^−2^ ±4.46*e*^−2^	1.00*e*^−1^ ±3.96*e*^−2^
n.^o^ 5	6.46*e*^−2^ ±3.20*e*^−2^	8.14*e*^−2^ ±2.58*e*^−2^

#### 6.6.2. Deep architectures

**Figure 7:**
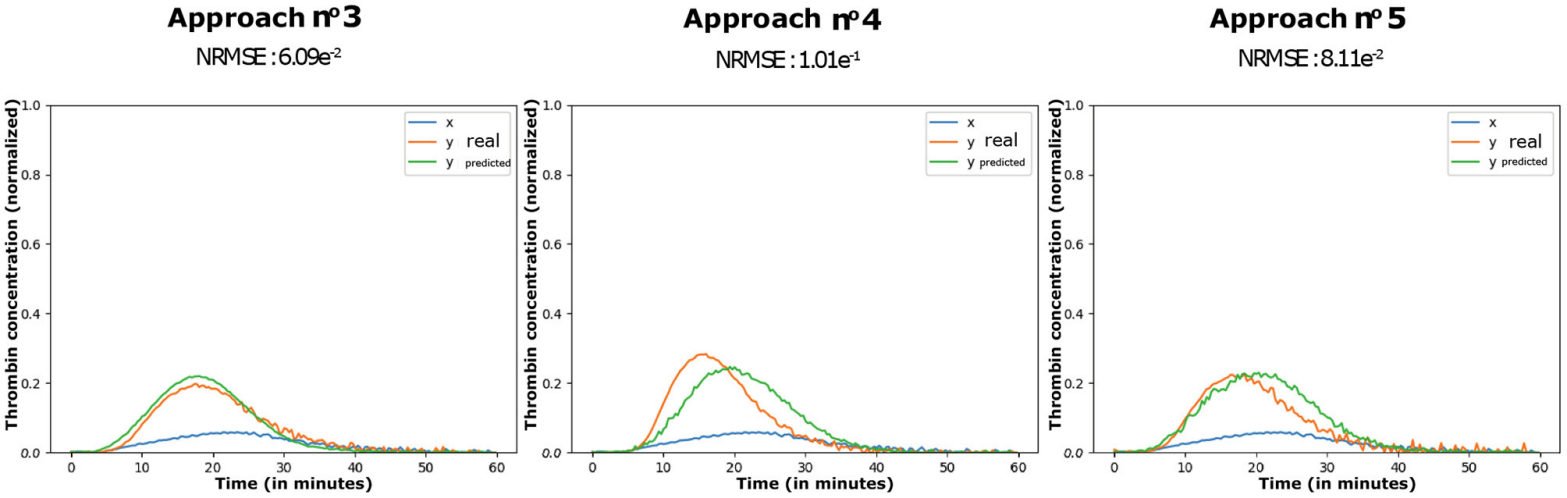
Representation of the error rates associated with Table 1.

The application of our proposed conditional incremental optimization strategy (approach n.^o^ 3), based on *in vitro* data, lasted 1,176 s on average. From the ten retries, a total of 304 DCAEs were optimized by approach n.^o^ 4 and 101 DCAEs by approach n.^o^ 5. The associated reconstruction error rates (NRMSE) are shown in Table 1 and illustrated in Figure 7.

Moreover, despite the low standard deviations of the error rates obtained by approach n.^o^ 3, four types of final topologies resulted from the dynamic optimization of the parameters and hyperparameters. Their architecture is detailed in Table 2.

**Table 2:** DCAE architectures obtained by approach n.^o^ 3 on the *in vitro* database.

	**Sub-architecture associated with:**		
	*p*(*x*)	*p*(*h*_*y*_|*h*_*x*_, *c*)	*p*(*y*)
A	*x* → *h*_*x*1_ → *h*_*x*2_ → *h*_*x*_ → ĥ_*x*2_ → ĥ_*x*1_ → *r*_*x*_	(*h*_*x*_, *c*) → *h*_*y*_	*h*_*y*_ → *h*_*y*1_ → *h*_*y*2_ → *y*
B	*x* → *h*_*x*1_ → *h*_*x*2_ → *h*_*x*_ → ĥ_*x*2_ → ĥ_*x*1_ → *r*_*x*_	(*h*_*x*_, *c*) → *h*_*y*_	*h*_*y*_ → *h*_*y*1_ → *h*_*y*2_ → *h*_*y*3_ → *y*
C	*x* → *h*_*x*1_ → *h*_*x*2_ → *h*_*x*3_ → *h*_*x*_ → ĥ_*x*3_ → ĥ_*x*2_ → ĥ_*x*1_ → *r*_*x*_	(*h*_*x*_, *c*) → *h*_*y*_	*h*_*y*_ → *h*_*y*1_ → *h*_*y*2_ → *y*
D	*x* → *h*_*x*1_ → *h*_*x*2_ → *h*_*x*3_ → *h*_*x*_ → ĥ_*x*3_ → ĥ_*x*2_ → ĥ_*x*1_ → *r*_*x*_	(*h*_*x*_, *c*) → *h*_*y*_	*h*_*y*_ → *h*_*y*1_ → *h*_*y*2_ → *h*_*y*3_ → *y*

### 6.7. Discussion

**Figure 8:**
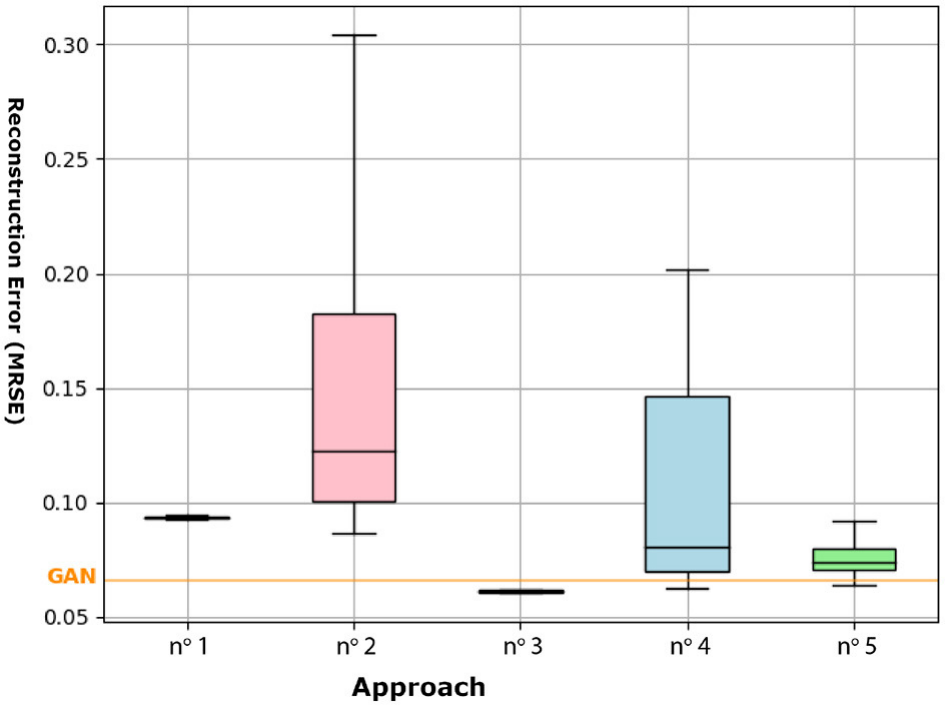
Box plot summarizing the error rates obtained by approaches n.^o^ 1 to 5 on the *in vitro* database. The orange horizontal line indicates the performance of the GAN (NRMSE = 0.066).

In Figure 8, the means and standard deviations of the error rates are lower in the case of the incremental CAE (approach n.^o^ 1) than in the random search (approach n.^o^ 2). In addition to the fact that this low variance underlines the robustness of our incremental building blocks to their initial topology (parameter values and learning rates), it highlights their regularization property and their interest for parametric and hyperparametric optimization of simple conditional architectures. Regarding the DCAEs, we can first note the interest of our proposed conditional pre-training (approach n.^o^ 5) which, by its regularization effect, also allows us to reduce the combinatorial complexity of this operational research. The application of our conditional incremental optimization strategy (approach n.^o^ 3), whose operating principle is based on the combination of the first two proposals, allows us to override the performance of the GAN and shows strong repeatability. In view of these results, we are able to address the difficulties of parametric and hyperparametric optimization of DCAEs.

## 7. Conclusion and future work

Therapeutic individualization is a method that entails the use of biological assays close to physiological reality. In the context of blood coagulation, routine laboratory tests only provide a partial assessment of the formation of a blood clot, whereas global assays have proved to be promising contenders in improving personalized care for patients suffering from hemostasis disorders. However, despite their relevance, these tests lack standardization, and their results have proved difficult for non-specialized clinicians to interpret.

In order to fully exploit the predictive behavior of global assay results, highly abstract characteristics can be extracted through deep architectures. In this respect, we have provided solutions to the challenge of both parametric and hyperparametric optimization of deep autoencoders. These solutions, characterized by a conditional pre-training algorithm and incremental optimization strategies, reduce the variance of the estimation process and enhance the convergence of the learning algorithm. Applying these solutions in the context of personalized care of hemophiliac patients, therefore, makes it possible to exceed the performance of generative adversarial networks and highlights the benefits of AE.

Since any *f*:ℝ^*n*^ → ℝ^*n*^ function where *n* is a finite value can be considered as a forward propagating neural network (Goodfellow et al., 2016), we have found the answer to our problem by omitting the temporal aspect (static approach). Nevertheless, it could be interesting to further develop our biological proposal by employing models specialized in the processing of sequential data, such as recurrent neural networks (Rumelhart et al., 1986; Hochreiter and Schmidhuber, 1997). Through a very deep computational graph, these models share the same parameters for all input features. They are then able to process data of variable size and to overcome the difficulties of phase shift that we encountered (Goodfellow et al., 2016). With this same objective in mind, we could also consider enriching the architecture by adding a temporal memory (Lasson et al., 2017). Combined with a sliding window principle, this approach would also allow the network parameters to be shared among all the input characteristics. We could then take into account the temporal aspect of kinetics while benefiting from the proposed optimization strategies.

## Data availability statement

The original contributions presented in the study are included in the article/supplementary material, further inquiries can be directed to the corresponding author.

## Author contributions

CB, FL, and SK contributed to conception and design of the study. FL developed the software and performed the statistical analyzes. CB and SK were the project administrators and supervised the work. All authors contributed to manuscript revision, read, and approved the submitted version.

## Footnotes

^1^The minimum and maximum values of the learning rates are optimized by a grid search during the initialization phase of the topology such that $\left(lr_{min},lr_{max}\right)=\left\{\left(5e^{-4},1e^{-3}\right),\left(1e^{-3},5e^{-3}\right),\left(5e^{-3},1e^{-2}\right)\right\}$. The initial width was defined as *L* = 360, i.e., twice the dimension of the input data.

^2^The hyperparameters are randomly selected from the following sets: width of the inner layer *L* ∈ [1, 360], learning rate *lr* ∈ {1*e*^−2^; 5*e*^−3^; 1*e*^−3^; 5*e*^−4^} and sparsity penalty λ ∈ {1*e*^−3^; 1*e*^−4^; 1*e*^−5^}.

^3^The depths of the networks associated with *p*(*x*) and *p*(*y*) are randomly defined between 1 and 4 hidden layers. The widths of the hidden layers are randomly specified in the range *L* ∈ [1, 360]. A decreasing sequencing for *p*(*x*) and increasing one for *p*(*y*) is nevertheless ensured. The learning rates required for the gradient descent algorithm and pre-training are selected from the set *lr* ∈ {1*e*^−2^; 5*e*^−3^; 1*e*^−3^; 5*e*^−4^}. The sparsity penalty is equal to λ ∈ {1*e*^−3^; 1*e*^−4^; 1*e*^−5^}.

^4^In order to optimize the depth of these two networks (*L* ∈ [1, 3]), their widths as well as their activation functions (sigmoidal units or hyperbolic tangents), various grid searches were performed. Taking into account the retries, 663 configurations were evaluated.

## References

[B1] Ashfahani A. Pratama M. Lughofer E. Ong Y.-S. (2020). DEVDAN: deep evolving denoising autoencoder. Neurocomputing 390, 297–314. 10.1016/j.neucom.2019.07.106

[B2] Bengio Y. (2009). Learning deep architectures for ai. Found. Trends Mach. Learn. 2, 1–127. 10.1561/2200000006

[B3] Bengio Y. Pascal L. Dan P. Larochelle H. (2007). Greedy layer-wise training of deep networks, in Advances in Neural Information Processing Systems 19, eds Schölkopf P. B. Platt J. C. Hoffman T. (MIT Press).

[B4] Blier L. Wolinski P. Ollivier Y. (2018). Learning with random learning rates. arXiv:arXiv[Preprint].1810.01322. 10.48550/arXiv.1810.01322

[B5] Erhan D. Bengio Y. Courville A. Manzagol P.-A. Vincent P. Bengio S. (2010). Why does unsupervised pre-training help deep learning? J. Mach. Learn. Res. 11, 625–660. Available online at: https://www.jmlr.org/papers/v11/erhan10a.html

[B6] Fahlman S. E. Lebiere C. (1990). The cascade-correlation learning architecture, in Advances in Neural Information Processing Systems 2, ed Touretzky D. S. (Morgan-Kaufmann), 524–532. Available online at: http://papers.nips.cc/paper/207-the-cascade-correlation-learning-architecture.pdf

[B7] Frey B. Dueck D. (2007). Clustering by passing messages between data points. Science 315, 972–976. 10.1126/science.1136800 17218491

[B8] Géron A. (2017). Deep Learning avec TensorFlow. Hors Collection. Dunod. Available online at: https://books.google.fr/books?id=Aa0-DwAAQBAJ

[B9] Glorot X. Bengio Y. (2010). Understanding the difficulty of training deep feedforward neural networks, in Proceedings of the Thirteenth International Conference on Artificial Intelligence and Statistics, volume 9 of Proceedings of Machine Learning Research (Sardinia: PMLR), 249–256.

[B10] Goodfellow I. Bengio Y. Courville A. (2016). Deep Learning. MIT Press. Available online at: http://www.deeplearningbook.org

[B11] Harkanen M. Vehvilainen-Julkunen K. Murrells T. Rafferty A. M. Franklin B. D. (2019). Medication administration errors and mortality: Incidents reported in england and wales between 2007–2016. Res. Soc. Admin. Pharm. 15, 858–863. 10.1016/j.sapharm.2018.11.010 30528260

[B12] Hinton G. E. Osindero S. Teh Y. W. (2006). A fast learning algorithm for deep belief nets. Neural Comput. 18, 1527–1554. 10.1162/neco.2006.18.7.1527 16764513

[B13] Hinton G. E. Salakhutdinov R. R. (2006). Reducing the dimensionality of data with neural networks. Science 313, 504–507. 10.1126/science.1127647 16873662

[B14] Hochreiter S. Schmidhuber J. (1997). Long short-term memory. Neural Comput. 9, 1735–1780. 10.1162/neco.1997.9.8.1735 9377276

[B15] Kawamoto K. Houlihan C. A. Balas E. A. Lobach D. F. (2005). Improving clinical practice using clinical decision support systems: a systematic review of trials to identify features critical to success. BMJ 330, 765. 10.1136/bmj.38398.500764.8F 15767266 PMC555881

[B16] Kingma D. P. Rezende D. J. Mohammed S. Welling M. (2014). Semi-supervised learning with deep generative models, in Advances in Neural Information Processing Systems 27, eds Ghahramani Z. Welling M. Cortes C. Lawrence N. D. Weinberger K. Q. (Curran Associates), 3581–3589. Available online at: http://papers.nips.cc/paper/5352-semi-supervised-learningwith-deep-generative-models.pdf

[B17] Lasson F. (2020). Intérêts des Auto-Encodeurs profonds pour les systèmes d'ide à l'individualisation de thérapies (Ph.D. thesis). ENIB.

[B18] Lasson F. Delamarre A. Redou P. Buche C. (2019). A clinical decision support system to help the interpretation of laboratory results and to elaborate a clinical diagnosis in blood coagulation domain, in International Work-Conference on Artificial Neural Networks (IWANN) (Gran Canaria), 109–122.

[B19] Lasson F. Polceanu M. Buche C. De Loor P. (2017). Temporal deep belief network for online human motion recognition, in 30th International Florida Artificial Intelligence Research Society Conference (FLAIRS) (Marco Island, FL), 80–85.

[B20] LeCun Y. Bengio Y. Hinton G. E. (2015). Deep learning. Nature 521, 436–444. 10.1038/nature14539 26017442

[B21] Lecun Y. Jdenker J. S. Solla S. A. (1990). Optimal brain damage, in Advances in Neural Information Processing Systems 2, ed Touretzky D. S. (Morgan Kaufmann), 598–605.

[B22] Lecun Y. Soulie Fogelman F. (1987). Modeles connexionnistes de l'apprentissage. Intellectica 2, 1804. 10.3406/intel.1987.1804

[B23] Makary M. A. Daniel M. (2016). Medical error – the third leading cause of death in the us. BMJ 353, i2139. 10.1136/bmj.i2139 27143499

[B24] Makhzani A. Shlens J. Jaitly N. Goodfellow I. Frey B. (2016). Adversarial autoencoders. arXiv 1511.05644. 10.48550/arXiv.1511.05644

[B25] Mirza M. Osindero S. (2014). Conditional generative adversarial nets. arXiv 1411.1784. 10.48550/arXiv.1411.1784

[B26] Ng A. (2017). Lecture notes. CS 294a: Sparse autoencoder. Technical report, Stanford University, Stanford, CA.

[B27] Pratama M. Ashfahani A. Ong Y. S. Ramasamy S. Lughofer E. (2018). Autonomous deep learning: Incremental learning of denoising autoencoder for evolving data streams. arXiv, abs/1809.09081. 10.48550/arXiv.1809.09081

[B28] Prechelt L. (1996). Early stopping-but when?, in Neural Networks: Tricks of the Trade, ed Orr G. B. Muller K. -R. (Springer), 55–69.

[B29] Reed R. (1993). Pruning algorithms-a survey. IEEE Trans. Neural Netw. 4, 740–747. 10.1109/72.248452 18276504

[B30] Rifai S. Vincent P. Muller X. Glorot X. Bengio Y. (2011). Contractive auto-encoders: Explicit invariance during feature extraction, in Proceedings of the 28th International Conference on International Conference on Machine Learning (Omnipress), 833–840.

[B31] Rumelhart D. E. Hinton G. E. Williams R. J. (1986). Learning representations by back-propagating errors. Nature 323, 533–536. 10.1038/323533a0

[B32] Sheikh A. Dhingra-Kumar N. Kelley E. Kieny M. Donaldson L. (2017). The third global patient safety challenge: tackling medication-related harm. Bull. World Health Organ. 95, 546. 10.2471/BLT.17.198002 28804162 PMC5537758

[B33] Sohn K. Yan X. Lee H. (2015). Learning structured output representation using deep conditional generative models, in Proceedings of the 28th International Conference on Neural Information Processing Systems (Montreal, QC: MIT Press), 3483–3491. Available online at: http://dl.acm.org/citation.cfm?id=2969442.2969628

[B34] Vincent P. Larochelle H. Bengio Y. Manzagol P. -A. (2008). Extracting and composing robust features with denoising autoencoders, in International Conference on Machine Learning Proceedings.

[B35] Zhou G. Sohn K. Lee H. (2012a). Online incremental feature learning with denoising autoencoders, in Proceedings of the Fifteenth International Conference on Artificial Intelligence and Statistics, volume 22 of Proceedings of Machine Learning Research, eds Lawrence N. D. Girolami M. (La Palma, CA: PMLR), 1453–1461.

[B36] Zhou G. Sohn K. Lee H. (2012b). Supplementary material: Online incremental feature learning with denoising autoencoders. J. Mach. Learn. Res. 22, 1453–1461. Available online at: https://www.researchgate.net/publication/265541462_Supplementary_Material_Online_Incremental_Feature_Learning_with_Denoising_Autoencoders

